# FGF23 as a Potential Pathophysiological Factor in Peripheral Arterial Disease Associated with Chronic Kidney Disease

**DOI:** 10.3390/ijms25105457

**Published:** 2024-05-17

**Authors:** Javier Donate-Correa, Ernesto Martín-Núñez, Carolina Hernández-Carballo, Ainhoa González-Luis, Carmen Mora-Fernández, Alberto Martín-Olivera, Sergio Rodríguez-Ramos, Purificación Cerro-López, Ángel López-Castillo, Alejandro Delgado-Molinos, Victoria Castro López-Tarruella, Juan F. Navarro-González

**Affiliations:** 1 Research Unit, University Hospital Nuestra Señora de Candelaria (UHNSC), 38010 Santa Cruz de Tenerife, Spain; emarnu87@gmail.com (E.M.-N.); carolinahdezcarballo@gmail.com (C.H.-C.); ainhoa.gonaluz@gmail.com (A.G.-L.); carmenmora.fdez@gmail.com (C.M.-F.); olimarabe@gmail.com (A.M.-O.); 2 GEENDIAB (Grupo Español Para el Estudio de la Nefropatía Diabética), Sociedad Española de Nefrología, 39000 Santander, Spain; 3 Instituto de Tecnologías Biomédicas, Universidad de La Laguna, 38000 Santa Cruz de Tenerife, Spain; 4 RICORS2040 (RD21/0005/0013), Instituto de Salud Carlos III, 28000 Madrid, Spain; 5 Navarrabiomed (Miguel Servet Foundation), Hospital Universitario de Navarra (HUN), Universidad Pública de Navarra (UPNA), 31008 Pamplona, Spain; 6 Doctoral and Graduate School, University of La Laguna, 38200 San Cristóbal de La Laguna, Spain; 7 Transplant Coordination, Hospital Universitario Nuestra Señora de Candelaria, 38010 Santa Cruz de Tenerife, Spain; sergiotomasr@hotmail.com (S.R.-R.); pcerlop@gobiernodecanarias.org (P.C.-L.); 8 Vascular Surgery Service, Hospital Universitario Nuestra Señora de Candelaria, 38010 Santa Cruz de Tenerife, Spain; angellopezcastillo24@gmail.com (Á.L.-C.); adelgadomolinos@gmail.com (A.D.-M.); 9 Pathological Anatomy Service, Hospital Universitario Nuestra Señora de Candelaria, 38010 Santa Cruz de Tenerife, Spain; vcaslop@gmail.com; 10 Nephrology Service, University Hospital Nuestra Señora de Candelaria, 38010 Santa Cruz de Tenerife, Spain; 11 Facultad de Ciencias de la Salud, Universidad Fernando Pessoa Canarias, 35450 Las Palmas de Gran Canaria, Spain

**Keywords:** chronic kidney disease, peripheral artery disease, inflammation, fibroblast growth factor 23, atherosclerosis

## Abstract

Fibroblast growth factor 23 (FGF23) levels are often elevated in chronic kidney disease (CKD). FGF23 and inflammation are common characteristics in CKD, and both are associated with worse disease progression and the occurrence of complications. The existence of an interaction between FGF23 and inflammation has been suggested, each of which influences the expression and activity of the other, leading to a vicious feedback loop with adverse outcomes, including cardiovascular disease and mortality. In this work, we determined circulating FGF23 levels in a group of patients with CKD stages 3 and 4 subjected to elective femoral endarterectomy due to established peripheral artery disease (PAD), a condition resulting from an athero-inflammatory process, and we studied its associations with different inflammatory markers and mediators. We evaluated its association with serum tumor necrosis factor (TNF)α, interleukin (IL) 6, and IL10, as well as with the gene expression levels of these parameters and A disintegrin and metalloproteinase domain-containing protein (ADAM) 17 in femoral vascular tissue and peripheral blood circulating cells (PBCCs). We also analyzed its association with serum concentrations of C-reactive protein (CRP), the systemic immune inflammation index (SII), and the neutrophil-to-lymphocyte ratio (NLR). Finally, we determined the vascular immunoreactivity of protein TNFα in a subgroup of patients. FGF23 concentrations were independently associated with circulating and PBCC mRNA levels of TNFα. Worst kidney function and diabetes were also found to be contributing to FGF23 levels. Patients with higher levels of FGF23 also had greater vascular immunoreactivity for TNFα.

## 1. Introduction

Patients with chronic kidney disease (CKD) are at high risk of developing atherosclerotic disease and related adverse outcomes [1,2]. Inflammation is a key factor in the pathophysiology of CKD and a potential contributor to the atherosclerotic risk [3,4]. A clinical manifestation of atherosclerotic disease commonly present in patients with CKD is peripheral arterial disease (PAD) [4]. The incidence of PAD in this population is strongly associated with lower glomerular filtration rate (GFR) and albuminuria [5]. PAD is closely related to systemic inflammation and constitutes the main cause of nontraumatic leg amputation and an independent risk factor for other cardiovascular diseases such as myocardial infarction [6,7].

Diverse pro-inflammatory mediators, such as interleukins (IL1 and IL6), tumor necrosis factor (TNF)α, and C-reactive protein (CRP), are increased in CKD patients and participate in the atherogenic process [8,9]. In addition to these factors, recent evidence supports the potential involvement of fibroblast growth factor 23 (FGF23) in the development of atherosclerosis and cardiovascular disease (CVD), showing strong associations with endothelial dysfunction, left ventricular hypertrophy, coronary artery disease, and cardiovascular mortality in CKD [10,11,12,13], as well as with vascular dysfunction and total body atherosclerosis [14,15]. FGF23 is a bone-derived hormone involved in the regulation of phosphorus homeostasis, vitamin D metabolism, and bone mineralization. Specifically, FGF23 inhibits the activation of vitamin D, induces phosphaturia, and suppresses parathyroid hormone (PTH) synthesis [16,17,18]. Elevated FGF23 levels are prevalent in kidney patients and are associated with poor outcomes and complications in both CKD and end-stage renal disease (ESRD) patients [19]. Importantly, higher levels of FGF23 are independently associated with inflammatory markers in CKD patients and with significantly higher odds of severe inflammation [20]. The expression of FGF23 in bone is regulated by the levels of phosphate, vitamin D, and PTH, as has been recently demonstrated, as well as by inflammatory cytokines [21,22]. Moreover, experimental data indicate that FGF23 can directly promote the synthesis of inflammatory factors, closing this potentially harmful feedback loop [23,24].

Elevated levels of FGF23 have been associated with the presence and severity of PAD in the population with diabetes mellitus (DM) and preserved kidney function [24]. However, there are limited data on the relationship between FGF23 levels and those of circulating and vascular inflammatory mediators in patients with CKD and PAD. In the present study, we determined the levels of serum FGF23 in a group of nondialyzed patients with CKD stages 3 and 4 subjected to elective femoral endarterectomy due to established clinical PAD and studied its correlation with different inflammatory markers. To do this, we determined the serum concentrations of TNFα, IL6, and IL10, and the gene expression levels of these parameters and A disintegrin and metalloproteinase domain-containing protein (ADAM) 17, also known as TNFα converting enzyme (TACE), in femoral vascular tissue and peripheral blood circulating cells (PBCCs). We also determined the vascular immunoreactivity of protein TNFα in a small subgroup of patients. The values of the inflammatory markers C-reactive protein (CRP), systemic immune inflammation index (SII), and neutrophil-to-lymphocyte ratio (NLR) were also determined.

## 2. Results

For this study, 150 patients were considered for enrollment, and 84 were excluded due to the exclusion criteria. Therefore, 66 patients (46 male, 69.7%) with a mean age of 63.1 ± 8.3 years and a body mass index (BMI) of 28.8 ± 3.9 kg/m^2^ were finally included (Table 1). Twenty-eight subjects (42.4%) had DM. Most of the patients had CKD stage 3 (50, 75.8%), and the mean GFR was 41.9 ± 12.4 mL/min/1.73 m^2^. Macroalbuminuria (urinary albumin-to-creatinine ratio (ACR) > 300 mg/g) was present in 15 patients (22.7%).

About half of patients with DM were under insulin therapy (57.1%). The percentage of current smokers and dyslipidemia was 34.8% and 74.2%, respectively. No patient received treatment with sodium–glucose cotransporter-2 inhibitors, nonsteroidal mineralocorticoid receptor antagonists, or glucagon-like peptide-1 receptor agonists.

The levels of FGF23 and inflammatory cytokines determined in serum, PBCCs, and vascular tissues are presented in Table 2, together with NLR, SII, and hs-CRP values. The logarithmic-transformed mean serum FGF23 value was 1.73 ± 0.19 pg/mL. This value was higher in patients with DM (1.83 ± 0.18 vs. 1.62 ± 0.15 pg/mL; *p* < 0.001). No differences were observed in serum FGF23 levels considering the smoking habits in patients with or without diabetes.

**Figure 1:**
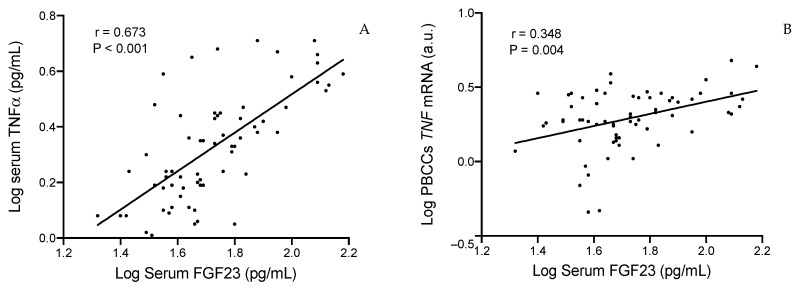
Correlations between serum FGF23 values with serum TNFα (**A**) and PBCCs *TNF* mRNA (**B**). FGF23, fibroblast growth factor 23; TNFα, tumor necrosis factor alpha; PBCCs, peripheral blood circulating cells. N = 66.

We performed bivariate correlation analyses to determine the associations of FGF23 with different variables (Table 3 and Figure 1). Serum FGF23 concentration was associated with worse kidney function and inversely related to eGFR (r = −0.576, *p* < 0.001) and directly to ACR (r = 0.439, *p* < 0.001). Regarding inflammatory parameters, FGF23 was only related to circulating levels of TNFα (r = 0.673, *p* < 0.001). Interestingly, HbA1c concentration was also directly related to FGF23 levels (r = 0.446, *p* < 0.001).

**Figure 2:**
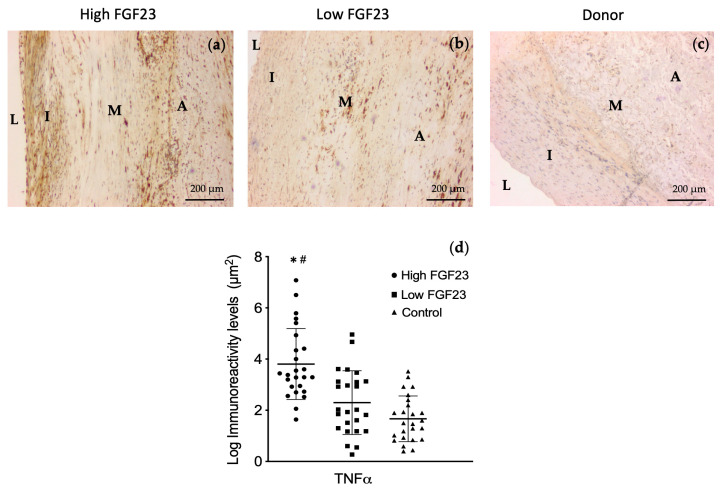
Representative images of femoral arterial sections from patients included in the high-FGF23 (**a**), how-FGF23 (**b**), and donor (**c**) groups. Scatter plot graph representing mean values and standard deviations (**d**). N = 5 patients in each group. High FGF23 > 48.5 pg/mL; low FGF23 < 48.5 pg/mL; original magnification 100×. L, lumen; I, tunica intima; M, tunica media; A, adventitia. * *p* < 0.01 vs. low-FGF23 group; # *p* < 0.01 vs. control group.

Immunohistochemical determinations of TNFα were made in sections of arterial territories obtained from 10 surgical PAD patients, namely 5 from patients with FGF23 serum levels above the median values in the whole population (high-FGF23 group ≥ 48.5 pg/mL) and 5 below this threshold (low-FGF23 group < 48.5 pg/mL). There were no significant differences in age (61.2 ± 8.1 vs. 64.2 ± 11.1 years), mean eGFR (43.7 [33.8–55.7] vs. 47.1 [31.3–52.9] mL/min/1.73 m^2^), ACR (38.1 [21.7–194] vs. 45.9 [27.1–210]), sex (60% male, for both), HbA1c (6.01 [5.3–7.2] vs. 6.21 [5.8–7.1]), or prevalence of DM (46% vs. 51%) and hypertension (80% for both) between these groups. For comparative purposes, arterial samples were also obtained from five cadaveric organ donors (mean age 51 ± 9 years; 80% men; nondiabetics and preserved kidney function -eGFR ≥90 mL/min/1.73 m^2^ and ACR <30 mg/g-). Image analysis revealed that the mean immunoreactivity levels for TNFα were significantly higher in vascular sections of the group with higher serum FGF23 levels when compared to sections from patients in the low-FGF23 group and the donor group (3.9 ± 1.4 vs. 2.3 ± 1.25 vs. 1.67 ± 0.89 log µm^2^, respectively; *p* < 0.01 for both comparisons) (Figure 2).

Finally, to determine independent associations with FGF23, we performed a backward stepwise multiple regression analysis with FGF23 as the dependent variable and diverse potential predictors, including, among others, BMI, eGFR, ACR, serum TNFα, PBCCs mRNA TNF, HbA1c, and DM. The results showed that only lower eGFR, higher ACR values, DM, and TNFα serum and PBCC expression levels were independently associated with increased values of FGF23 in these patients (adjusted R^2^ = 0.722, *p* < 0.001) (Table 4).

## 3. Discussion

The main finding of the present study is that, in patients with CKD and PAD, FGF23 levels are independently associated with circulating TNFα concentrations and mRNA expression levels of this cytokine in PBCCs. Worse kidney function and DM also appeared as contributors to FGF23 levels. More striking, the results of the immunohistochemical study performed in the vascular tissue showed an increase in TNFα in those subjects with higher levels of circulating FGF23. We found no associations of FGF23 with systemic inflammatory markers, including SII, hs-CRP, or NLR, nor with the levels of the inflammatory cytokines IL6, IL10, or ADAM17.

Increased levels of FGF23 have been associated with endothelial dysfunction, arterial wall calcification, left ventricular hypertrophy, coronary artery disease, unstable carotid atherosclerosis, and cardiovascular mortality [13,14,15,19,24]. Elevated levels of FGF23 have also been associated with the presence and severity of PAD in patients with DM and preserved kidney function [24]. Larger population studies have also related higher FGF23 concentrations with the incidence of PAD events even after adjustment for different demographic and CVD risk factors, as well as for eGFR and ACR [25]. Similarly, in the Homocysteine in Kidney and End-Stage Renal Disease (HOST) study, subjects in the highest quartile of FGF23 had a significantly higher risk of amputation due to PAD [26]. HOST participants had a mean age of 69 years and severely reduced kidney function (mean eGFR 18 mL/min/1.73 m2) and therefore older and with more advanced kidney disease than those in our study (mean age 63.1 ± 8.3 yrs and mean eGFR 41.9 ± 12.4 mL/min/1.73 m^2^).

Although the effects of FGF23 on the immune system are mostly unknown, recent studies point to the existence of a feedback mechanism between FGF23 and several inflammatory modulators. The results of the Chronic Renal Insufficiency Cohort (CRIC) study showed that higher FGF23 levels are associated with higher levels of the inflammatory markers CRP, IL6, TNFα, and fibrinogen, and with a greater odds ratio for severe inflammation independently of mineral metabolism and kidney function [20]. Several experimental studies suggest that inflammation is a major trigger for FGF23 production [21,27,28,29]. The exact mechanisms by which inflammation stimulates FGF23 production are still being studied, but it is likely that inflammatory cytokines TNFα and IL6 play a role in this process by directly stimulating FGF23 production or indirectly affecting it through various signaling pathways [21,30]. Conversely, experimental works also demonstrated that FGF23 is able to directly or indirectly activate the transcription of inflammatory regulators. Thus, FGF23 is able to inhibit calcitriol production in human PBCCs, which may indirectly contribute to the inflammatory state since calcitriol is a modulator of the immune system [31]. Moreover, experimental data indicate that FGF23 can directly promote the synthesis of regulatory inflammation genes such as TGFß1, TNF, and IL1ß [32]. Similarly, FGF23 increases the expression of CRP and IL6 in hepatocytes [23]. The stimulation of IL6 and TNFα production elicited by FGF23 is abolished in diabetic nephropathy mouse models injected with the carboxy-tail peptide of FGF23, which prevents iFGF23 signaling [33]. Taken together, these results suggest a role of FGF23 in the modulation of the inflammatory response, although the regulatory mechanism and its physiological function are barely understood. Our results deepen the study of this association in patients with CKD and PAD, showing significant differences in the levels of circulating and vascular TNFα depending on the concentration of FGF23. Interestingly, patients with higher serum levels of FGF23 show significantly greater TNFα immunoreactivity levels in the femoral vascular tissue.

Another interesting result of our study is the correlation between FGF23 levels and HbA1c concentrations. DM is the most common cause of end CKD and is frequently associated with CV complications, compromising the quality of life of these patients and increasing the premature mortality rate [34]. PAD is a common complication in patients with DM and is in general more aggressive and with a higher incidence of tissue loss and amputations [35]. Observational studies show higher circulatory FGF23 levels in patients with DKD [36]. Moreover, FGF23 has been associated with obesity, dyslipidemia, visceral adiposity, insulin resistance, and an increased risk of metabolic syndrome [37]. It is not known whether the higher levels of FGF23 found in patients with diabetes and CKD may contribute to the worse clinical outcomes observed in these patients compared to subjects with CKD without DM [38].

Although presenting new interesting data, we acknowledge several limitations in this work. The small sample size limited the statistical power of the applied tests, so the findings may not be generalizable, and further studies including larger groups to confirm these observations are needed. A high proportion of our patients had DKD, which is associated with higher levels of FGF23. Furthermore, this high proportion may have limited our results, showing associations derived from processes that exclusively occur in patients with DKD. A high proportion of patients were prescribed statins, which have been shown to have anti-inflammatory effects. Serum concentrations of vitamin D and parathyroid hormone—potent stimulators of FGF23 production—were not measured, and therefore, their possible influence on the relationship between FGF23 and inflammation cannot be completely ruled out. Similarly, we did not determine the bioavailability/bioactivity of nitric oxide whose decrease contributes to endothelial dysfunction, an early event in the development of atherosclerosis. We also did not analyze the effects of various pharmacological therapies that may affect FGF23 levels, such as statins, renin–angiotensin system inhibitors, and insulin. Finally, given the cross-sectional design, our results show associations, but a causal relationship between FGF23 and inflammation cannot be definitely demonstrated.

Nevertheless, the aim of this study was to identify potential clinical associations of FGF23 with inflammatory parameters and modulators in patients with CKD and PAD. Although our findings are merely associative and do not demonstrate causality, they allow us to speculate about the role of imbalances in FGF23 levels on the inflammatory status in CKD patients and their pathophysiological implications in atherosclerotic vascular disease. Since inflammation is an important element in the development, progression, and prognosis of PAD in CKD, future studies should investigate the impact of interventions to reduce FGF23 on inflammatory markers and, conversely, the impact of anti-inflammatory therapies on FGF23 levels. Furthermore, the possible additive effects of combining such therapeutic strategies in improving clinical outcomes in CKD should also be determined.

In conclusion, our findings point to the existence of an independent association of FGF23 with serum, PBCCs, and vascular expression levels of TNFα in patients with CKD and PAD. DM also appeared as a contributing factor to FGF23 levels. Taken together, our results suggest that FGF23 could play a role in the inflammatory status of these patients with potential implications on the development and progression of PAD. The identification of novel pathophysiological pathways related to the PAD atherosclerotic process is important in further reducing its incidence since, despite the implementation of optimal medical therapy, a large number of patients with CKD still undergo revascularization and amputations due to this complication.

## 4. Materials and Methods

### 4.1. Study Design and Population

One hundred and fifty patients with CKD undergoing an elective femoral endarterectomy procedure between November 2014 and July 2016 at the Vascular Surgery Service of the University Hospital Nuestra Señora de Candelaria (UHNSC) due to established clinical PAD were considered for initial enrollment in this study. Exclusion criteria included hemodynamic instability during the surgical procedure; history of chronic inflammatory, immunologic, or tumoral disease; positive serology to hepatitis B, hepatitis C, or human immunodeficiency virus; acute inflammatory or intercurrent infectious episodes in the previous month; institutionalization; treatment with immunotherapy or immunosuppressive drugs; previous organ transplantation; ESRD; and inability or unwillingness to provide informed consent. The presence of established atherosclerotic vascular disease was confirmed with imaging studies that included computed tomography, magnetic resonance imaging, and catheter angiography. For comparative purposes in the immunohistochemistry study, femoral samples from 5 cadaveric organ donors with preserved kidney function and no clinical CVD were recovered during organ retrieval surgery. The study was conducted according to the Declaration of Helsinki and the guidelines of Good Clinical Practice and was approved by the local ethics committee of UHNSC (Ethic Committee Approval Code: PI-16/07). Written informed consent was obtained from all participants or their family members before participating in the study.

### 4.2. Blood and Vascular Tissue Determinations

Blood samples were drawn in the morning after an 8 h fast in routine blood collection tubes (BD Serum Separation Transport Tube, BD, Franklin Lakes, NJ, USA) and PAXgene blood RNA tubes (BD, Franklin Lakes, NJ, USA) to recover the serum fraction and for gene expression studies in PBCCs, respectively. Serum fractions were aliquoted and immediately frozen at −80 °C. Routine biochemical and hematological parameters were determined using standardized tests. Serum levels of intact FGF23 were measured using the Human FGF23 ELISA Kit (EMD Millipore Corporation, Milford, MA, USA), which has a sensitivity of 3.5 pg/mL and intra- and inter-assay coefficients of variation of 9.5% and 6.85%, respectively. Serum levels of the inflammatory cytokines TNFα, IL6, and IL10 were measured using commercial high-sensitivity ELISA methods (Quantikine^®^, R&D Systems, Abingdon, UK) with minimum detectable concentrations of 0.10 pg/mL, 0.70 pg/mL, and 0.50 pg/mL, respectively, and with intra- and inter-assay variability coefficients <10.8%. The SII was calculated according to the following formula: (platelets × neutrophils)/lymphocytes. The NLR was calculated as the simple ratio between the neutrophil and lymphocyte counts measured in peripheral blood. 

During vascular surgery, a sample of the femoral artery was obtained from all the participants. For immunohistochemical analysis of the vascular samples, recovered blood vessel fragments were fixed in 4% (*v*/*v*) buffered formalin for 24 h and subsequently dehydrated in ascending series of ethanol, cleared in xylene, and embedded in paraffin blocks. Trimmed 3 μm sections were processed for immunohistochemistry. Primary rabbit polyclonal anti-TNFα (Abcam ab6671, 1:100 dilution) was employed. Slides were counterstained with hematoxylin. For the quantification analysis, a total of 5 images of each slide, which included intima and media layers, were captured. Then, images were digitalized, and the area of tissue stained by the antibody was quantified by using ImageJ 1.53 software, which was free to download from the website of the National Institute of Health (NIH) (http://imagej.nih.gov/ij/, accessed on 1 November 2020) (Rasband, W.S., ImageJ, National Institutes of Health, Bethesda, MD, USA). The results are expressed in square microns.

Total RNA from blood samples was isolated using PAXgene Blood RNA Kit (PreAnalytiX, Hombrechtikon, Switzerland) following the manufacturer’s guidelines. Vascular RNA was extracted using RNAzol RT (Sigma-Aldrich, St. Loui, MO, USA) after homogenization in liquid nitrogen with a pestle and mortar. To carry out the gene expression study, blood and vascular RNA were retrotranscribed to cDNA using a high-capacity RNA-to-cDNA kit (Applied Biosystems, Foster City, CA, USA). Blood and vascular transcripts encoding for TNF, IL6, IL10, ADAM17, and glyceraldehyde 3-phosphate dehydrogenase (GAPDH) genes were measured by TaqMan real-time quantitative PCR (qRT-PCR) employing TaqMan Fast Universal PCR Master Mix (Thermo Fisher, Waltham, MA, USA) and the following TaqMan gene expression assays: Hs00174128_m1 (TNF), Hs00985639_ml (IL6), Hs0961622_m1 (IL10), Hs01041915_m1 [ADAM17], and Hs99999905_m1 (GAPDH). The level of target mRNA was estimated by relative quantification using the comparative method (2^−ΔΔCt^) by normalizing to GAPDH expression.

### 4.3. Statistical Analysis

Continuous variables are reported as mean ± standard deviation (SD) or medians and interquartile ranges (IQRs). Categorical data are presented as percentages. Continuous variables were assessed for normal distribution by D’Agostino–Pearson test and those variables that presented non-normal distribution were log-transformed for statistical analysis. Differences among groups were analyzed by unpaired *t*-test, Mann–Whitney test, or one-way analysis of variance with Tukey’s post hoc test. Categorical variables were compared between groups using Fisher’s exact test. Correlation analysis was evaluated by the Spearman correlation test. Multiple backward stepwise regression analysis was performed using FGF23 serum levels as the dependent variable. Values of *p* < 0.05 were considered significant. Statistical analyses were performed using IBM SPSS Statistics V.26 (IBM Corporation, Armonk, NY, USA) and GraphPad Prism V.9 software (GraphPad Software, San Diego, CA, USA).

## 5. Conclusions

FGF23 levels are often elevated in patients with CKD. FGF23 and inflammation are common characteristics in CKD, and both are associated with worse disease progression and the occurrence of complications. A clinical manifestation of atherosclerotic disease commonly present in patients with CKD is PAD. The results of the present study point to the existence of an independent association of FGF23 with TNFα serum and PBCC expression levels in patients with CKD and PAD. These findings may be of clinical relevance because provide new mechanistic insights about the pathophysiology of atherosclerosis disease in CKD. The interrelationship between FGF23 and inflammation opens new research pathways regarding the development and progression of atherosclerosis in subjects with CKD, with translational clinical applicability from diagnostic, prognostic, and therapeutic perspectives focused on modulating FGF23.

## Figures and Tables

**Table 1:** Demographic and biochemical characteristics of patients with PAD.

Characteristics	
N	66
Age (years)	63.15 ± 8.3
Sex (% male)	46 (69.7)
BMI (kg/m^2^)	28.8 ± 3.9
SBP (mm Hg)	131.6 ± 22.7
DBP (mm Hg)	68.4 ± 14.1
Comorbidities	
Diabetes mellitus type 2	28 (42.4)
Hypertension (%)	55 (83.3)
Current smokers (%)	21 (31.8)
Macroalbuminuria (%)	15 (22.7)
CKD Stage 3/4	50/16
Dyslipidemia (%)	49 (74.2)
Pharmacological treatment	
Antiplatelet (%)	55 (83.3)
Beta-blockers (%)	35 (53)
ACEI/ARA2 (%)	32 (48.5)
CCB (%)	23 (34.8)
Statins (%)	47 (71.2)
Insulin (%)	16 (24.2)
Laboratory data	
Hemoglobin (g/dL)	11.2 (9.9–12.8)
Creatinine (mg/dL)	1.56 (1.27–2.2)
eGFR (mL/min/1.73 m^2^)	41.9 (30.9–52.4)
ACR (mg/g)	36.9 (23–263)
T-cholesterol (mg/dL)	161.2 ± 47.8
HDL-C (mg/dL)	40.9 ± 13.2
LDL-C (mg/dL)	89.9 ± 36.8
Triglycerides (mg/dL)	144.7 ± 57.7
Fasting glucose (mg/dL)	123 (98–161)
HbA1c (%)	6.15 (5.5–7.6)
Neutrophils (×10^9^ cells/L)	7.53 ± 4.9
Lymphocytes (×10^9^ cells/L)	1.62 ± 0.79
Platelets (×10^9^ cells/L)	212 ± 83.6
Uric acid (mg/dL)	6.55 ± 1.8
Calcium (mg/dL)	9 (8.6–9.3)
Phosphorus (mg/dL)	3.84 ± 0.73

BMI, body mass index; SBP, systolic blood pressure; DBP, diastolic blood pressure; CKD, chronic kidney disease; ACEI, angiotensin-converting enzyme inhibitor; CCB, calcium channel blockers; eGFR, estimated glomerular filtration rate; ACR, urine albumin-to-creatinine ratio; HDL-C high-density lipoprotein cholesterol; LDL-C low-density lipoprotein cholesterol; Hb1ac, glycated hemoglobin.

**Table 2:** Determinations of FGF23 and biomarkers and mediators of inflammation.

Blood Determinations	
NLR	2.9 (2.16–9.26)
SII (×10^9^ cells/L)	875.9 (392.8–1480.9)
hs-CRP (mg/mL)	6.28 (2.4–11.14)
Log FGF23 (pg/mL)	1.73 ± 0.19
Log TNFα (pg/mL)	0.31 ± 0.22
Log IL6 (pg/mL)	1.03 ± 0.7
Log IL10 (pg/mL)	0.55 ± 0.85
PBCCs mRNA (a.u.)	
*TNF*	2.02 (1.56–2.7)
*IL6*	1.68 (0.59–4.64)
*IL10*	0.41 (0.31–0.72)
*ADAM17*	1.75 (1.24–2.9)
Vascular mRNA (a.u.)	
*TNF*	1.29 (0.75–2.9)
*IL6*	1.5 (0.47–24.8)
*IL10*	0.1 (0.05–0.55)
*ADAM17*	1.23 (0.71–1.8)

NLR, neutrophil-to-lymphocyte ratio; SII, systemic immune inflammation index; hs-CRP, high sensitivity C-reactive protein; FGF23, fibroblast growth factor 23; TNFα, tumor necrosis factor alpha; IL, interleukin; ADAM17, A disintegrin and metalloproteinase domain-containing protein 17; PBCCs, peripheral blood circulating cells; a.u., arbitrary units.

**Table 3:** Bivariate correlations of FGF23 levels with inflammatory markers and mediators and with renal and glycemic-control-related parameters.

	r	*p*
General biochemical determinations		
Age (years)	−0.226	0.07
BMI (kg/m^2^)	0.304	0.01
eGFR (mL/min/1.73 m^2^)	−0.576	<0.001
ACR (mg/g)	0.439	<0.001
Inflammatory markers and mediators		
NLR	0.026	0.83
SII (×10^9^ cells/L)	0.065	0.61
hs-CRP (mg/mL)	0.06	0.63
Log serum TNFα (pg/mL)	0.673	<0.001
Log serum IL6 (pg/mL)	0.18	0.18
Log serum IL10 (pg/mL)	0.045	0.73
PBCC *TNF* mRNA (a.u.)	0.348	0.004
PBCC *IL6* mRNA (a.u.)	−0.081	0.54
PBCC *IL10* mRNA (a.u.)	−0.09	0.55
PBCC *ADAM17* mRNA (a.u.)	0.029	0.83
Vascular *TNF* mRNA (a.u.)	−0.071	0.59
Vascular *IL6* mRNA (a.u.)	0.067	0.61
Vascular *IL10* mRNA (a.u.)	0.027	0.84
Vascular *ADAM17* mRNA (a.u.)	0.012	0.93
Glycemic control parameters		
Fasting glucose (mg/dL)	−0.018	0.886
HbA1c (%)	0.446	<0.001

BMI, body mass index; eGFR, estimated glomerular filtrate rate; ACR, urine albumin-to-creatinine ratio; FGF23, fibroblast growth factor 23; NLR, neutrophil-to-lymphocyte ratio; SII, systemic immune inflammation index; hs-CRP, high-sensitivity C-reactive protein; TNFα, tumor necrosis factor alpha; IL, interleukin; PBCCs, peripheral blood circulating cells.

**Table 4:** Multiple backward stepwise regression with the serum levels of FGF23 as the dependent variable.

	Adjusted R^2^	ß	SE	t	*p*
Serum FGF23 (pg/mL)	0.779				<0.001
eGFR (mL/min/1.73 m^2^)		−0.285	0.001	−4.254	<0.001
ACR (mg/g)		0.178	3 × 10^−6^	2.942	0.005
Diabetes mellitus		0.348	0.024	5.593	<0.001
Log serum TNFα (pg/mL)		0.350	0.061	5.708	<0.001
PBCC *TNF* mRNA (a.u.)		0.245	0.015	4.003	<0.001

FGF23, fibroblast growth factor 23; eGFR, estimated glomerular filtrate rate; ACR, urine albumin-to-creatinine ratio; TNFα, tumor necrosis factor alpha.

## Data Availability

Queries related to data access should be directed to the corresponding authors.
